# The effects of intravenous anesthetics on QT interval during anesthetic induction with desflurane

**DOI:** 10.1186/s40981-018-0195-9

**Published:** 2018-07-30

**Authors:** Shozo Tominaga, Yoshiaki Terao, Shigehiko Urabe, Maki Ono, Natsuko Oji, Makito Oji, Makoto Fukusaki, Tetsuya Hara

**Affiliations:** 1 Department of Anesthesia, Nagasaki Rosai Hospital, 2-12-5 Setogoe, Sasebo, 857-0134 Japan; 2 Department of Anesthesiology, Nagasaki University School of Medicine, 1-7-1 Sakamoto, Nagasaki, 852-8501 Japan

**Keywords:** Propofol, Desflurane, Thiamylal, QT interval, QT dispersion

## Abstract

**Introduction:**

This study aimed to determine the effects of the interaction between intravenous anesthetics and desflurane on the QT interval.

**Methods:**

Fifty patients who underwent lumbar spine surgery were included. The patients received 3 μg/kg fentanyl and were randomly divided into two groups: group P patients received 1.5 mg/kg propofol and group T patients received 5 mg/kg thiamylal 2 min after fentanyl injection. All patients received rocuronium and desflurane (6% inhaled concentration) after loss of consciousness. Tracheal intubation was performed 3 min after rocuronium injection. Heart rate (HR), mean arterial pressure (MAP), bispectral index score (BIS), and the heart rate-corrected QT (QTc) interval on a 12-lead electrocardiograms were recorded before fentanyl injection (T1), 2 min after fentanyl injection (T2), 1 min after propofol or thiamylal injection (T3), immediately before intubation (T4), and 2 min after intubation (T5).

**Results:**

There were no significant intergroup differences in patient characteristics. BIS and MAP decreased after anesthesia induction in both groups. MAP values at T3, T4, and T5 in group T were higher than those in group P. HR did not change over time or differ between the groups. The QTc intervals at T4 and T5 in group T were longer than those at T1. In group P, the QTc interval at T3 was significantly shorter than that at T1. The QTc intervals at T3, T4, and T5 in group T were significantly longer than those in group P.

**Conclusions:**

A propofol injection could counteract the QTc interval prolongation during desflurane anesthesia induction.

**Trial registration:**

UMIN Clinical Trials Registry database reference number: UMIN000023707. This study was registered on August 21, 2016.

## Background

The QT interval of the electrocardiogram (ECG) represents the period of myocardial depolarization and repolarization. Heart-rate-corrected QT (QTc) interval prolongation is associated with life-threatening dysrhythmias, including a polymorphic ventricular tachycardia called torsade de pointes (TdP). Thus, it is important to determine whether the anesthetics used prolong the QTc interval. Volatile anesthetics, including desflurane, are known to prolong the QT interval significantly [1]. We previously reported that propofol injection counteracted the QTc interval prolongation associated with sevoflurane anesthesia induction [2]. Desflurane is not used as the sole agent for anesthesia induction because of the associated airway irritation. Although propofol is generally used as an induction agent before desflurane administration, it appears that the preventive effect of propofol on desflurane-induced QTc interval prolongation has not been previously examined.

QT dispersion (QTD), the difference between the maximum and minimum QT intervals on the 12-lead ECG, is considered a measure of left ventricular repolarization inhomogeneity, which could serve as an electrophysiological index for an increased risk of ventricular dysrhythmia [3]. Increased QTD is a sign of heterogeneous repolarization and possible arrhythmogenic re-entry [4]. Yildirim et al. have reported that sevoflurane, isoflurane, and desflurane prolong the QTc interval and QTc dispersion (QTcD) [1], while Silay et al. have found that sevoflurane and desflurane prolong the QTc interval but do not influence QTD [5]. Our previous study showed that bolus administration of propofol did not affect the QTcD [6].

The aim of this randomized, open-label, clinical study was to determine the effects of the interaction between intravenous anesthetics and desflurane on the QTc interval and QTcD during anesthesia induction.

## Methods

### Patients

This open-label randomized clinical trial included 50 American Society of Anesthesiologists physical status 1 or 2 patients aged 20–69 years who underwent elective lumbar spine surgery, including laminectomy, microscopic discectomy, microendoscopic discectomy, or spinal fusion, under general anesthesia between August 2016 and July 2017. The exclusion criteria included a medical history of ischemic heart disease, diabetes, asthma, preoperative electrocardiographic abnormalities, body mass index > 30 kg/m^2^, and preoperative medications known to prolong the QTc interval, including β-adrenergic antagonists, antiarrhythmic agents, and antihypertensive medications such as calcium antagonists, angiotensin-converting enzyme inhibitors, and angiotensin 2 receptor antagonists. None of the patients received any preanesthetic medication.

### Study protocol

Pulse oximetry, 3-lead electrocardiography, non-invasive blood pressure monitoring, and bispectral index measurement (BIS; A2000 BIS Monitoring System; Aspect Medical System, Natick, MA, USA) were performed as the standard procedures for patients receiving general anesthesia. The incidence of dysrhythmia was monitored by continuous recording of electrocardiographic lead II data. The 12-lead electrocardiography system was attached before anesthesia induction, and standard 12-lead electrocardiographic recordings were obtained at each pre-specified measurement time point with a computerized electrocardiographic recorder (model FX-7432; Fukuda Denshi, Tokyo, Japan) at a paper speed of 25 mm/s. The electrocardiographic data were also recorded digitally. QT intervals were measured using the developed software (QTD-1; Fukuda Denshi, Tokyo, Japan), which is programmed to detect the onset of the QRS complex and the end of the T wave [6, 7]. The QTc interval was calculated according to Fridericia’s formula [8] as follows:

$$ \mathrm{QTc}=\mathrm{QT}/\sqrt[3]{\mathrm{RR}} $$

The data were excluded from analysis in cases where the QT interval could not be reliably measured because of T-wave morphology, and a minimum of 6-lead data were considered necessary for the analysis. QTcD was defined as the difference between the maximum and minimum QTc interval values in all leads. The mean QTc interval was calculated from all available QTc interval values averaged over three consecutive cycles in all leads during the measurement period. An investigator who was blinded to the anesthetic agent examined and analyzed the ECGs.

Patients were randomly allocated to groups T and P (*n* = 25 each) by sealed envelope assignment. All patients received oxygen via a facemask at a flow rate of 5 L/min for 1 min prior to injection of 3 μg/kg fentanyl. The patients in group T received 5 mg/kg thiamylal, and those in group P received 1.5 mg/kg propofol 2 min after fentanyl injection. Intravenous rocuronium (0.6 mg/kg) and inhaled desflurane (6% inhaled concentration) were administered to all patients after loss of consciousness, and tracheal intubation was performed 3 min after rocuronium injection. The eyelash reflex was continuously monitored after loss of verbal response, and loss of consciousness was determined by loss of the eyelash reflex [9]. The respiratory rate was adjusted to maintain an end-tidal carbon dioxide partial pressure of 35 mmHg. The inhaled concentration (6%) of desflurane was maintained during the study period. Heart rate (HR), mean arterial pressure (MAP), BIS, end-tidal desflurane concentration (ETdes), and 12-lead electrocardiographic data were recorded immediately before fentanyl injection (baseline: T1), 2 min after fentanyl injection (T2), 1 min after propofol or thiamylal injection (immediately before desflurane administration: T3), 3 min after desflurane administration (immediately before tracheal intubation: T4), and 2 min after tracheal intubation (T5).

### Statistical analysis

The results are expressed as median (interquartile range). A 2-factorial repeated-measures analysis of variance was performed to analyze the interaction between time and the two groups. A post hoc comparison between groups at each time point and among the repeated measures in each group was performed using the Dunnett procedure, if appropriate. Continuous data for patient characteristics were analyzed using the Mann-Whitney *U* test. Dichotomous variables were analyzed with either the Fisher’s exact probability or chi-squared test. A *p* value of < 0.05 was considered statistically significant.

Sample size was determined on the basis of a previous study (SD, 23 ms) [6], which indicated that with 22 patients in each group, a power of 90% would be required to detect a difference of 23 ms in the mean QTc interval value between the two groups at a 5% significance level.

## Results

Fifty-three patients, 38 male and 15 female, were enrolled in the study. Of these, 3 patients (1 male patient from each group and 1 female patient from Group T) were excluded because their electrocardiographic data were wrongly entered in the software. Thus, 50 patients were included in the final analysis. None of the participants required vasopressors during the study. Table 1 shows the patient characteristics. There were no significant differences between the two groups. Atrial premature contraction was observed in a patient in group T. Table 2 shows the values for ETdes, BIS, and circulatory variables for both groups. There were no significant intergroup differences in ETdes. BIS and MAP significantly decreased after anesthesia induction in both groups. The MAP in group P was lower than that in group T after anesthesia induction. There were no significant differences in HR and QTcD at any recorded time point.

**Table 1:** Patient characteristics

Variables	Thiamylal group	Propofol group	*P* value
Patients	25	25	
ASA 1/2	14/11	17/8	0.56
Sex (male/female)	18/7	18/7	1
Age (years)	39 (34–51 [24–65])	45 (33–57 [26–68])	0.79
Height (cm)	168 (161–174 [146–183])	171 (162–175 [147–185])	0.55
Weight (kg)	63 (53–63 [49–83])	60 (56–69 [46–85])	0.95
BSA (m^2^)	1.70 (1.56–1.80 [1.40–2.01])	1.73 (1.60–1.88 [1.36–1.98])	0.68
Sodium (mmol l^−1^)	140 (139–142 [138–143])	141 (140–142 [139–144])	0.06
Potassium (mmol l^− 1^)	4.2 (4.0–4.3 [3.9–4.5])	4.1 (4.0–4.3 [3.6–4.9])	0.31
Calcium (mmol l^− 1^)	2.3 (2.2–2.3 [2.1–2.4])	2.3 (2.2–2.3 [2.1–2.4])	0.44
Hemoglobin (g dl^− 1^)	14.5 (13.6–15.5 [11.0–16.7])	14.4 (13.8–15.0 [12.2–16.4])	0.62

Values are number, median (IQR [range])

*ASA* American Society of Anesthesiologists physical status, *BSA* body surface area

**Table 2:** Comparison of selected variables in thiamylal group and propofol group

Variable	Group	T1	T2	T3	T4	T5
ETdes (vol%)	T				3.6 (3.1–3.8 [2.7–3.9])	4.5 (4.3–4.7 [4.0–5.0])
	P				3.3 (3.0–3.6 [2.8–3.9])	4.3 (4.0–4.6 [3.7–5.0])
BIS	T	97 (94–97 [92–98])	95 (92–97 [76–98])	45 (35–55 [21–78])*	61 (49–68 [39–76])*	57 (50–61 [39–76])*
	P	95 (90–97 [91–98])	95 (91–97 [87–98])	51 (45–66 [37–88])*	40 (34–46 [26–64])*	39 (35–44 [39–76])*
HR (min^− 1^)	T	69 (63–82 [46–103])	67 (58–83 [49–109])	73 (63–76 [59–101])	69 (64–73 [57–88])	68 (64–75 [52–112])
	P	70 (62–79 [53–108])	67 (61–74 [56–101])	64 (59–73 [55–101])	68 (58–76 [53–87])	66 (61–72 [49–96])
MAP (mmHg)	T	93 (86–104 [71–125])	95 (90–98 [81–142])	89 (82–99 [69–116])*	81 (72–88 [58–107])*	81 (74–90 [63–140])*
	P	94 (81–102 [74–139])	90 (83–96 [70–125])	76 (64–84 [59–102])^#^*	69 (62–78 [51–107])^#^*	71 (64–85 [56–112])^#^*
QTcD (ms)	T	52 (39–55 [24–74])	48 (42–62 [22–79])	44 (34–55 [24–80])	43 (34–56 [20–96])	44 (38–56 [22–72])
	P	40 (34–53 [29–57])	41 (32–58 [28–90])	48 (42–62 [20–82])	39 (32–48 [22–75])	40 (31–45 [24–64])

Values are median (IQR [range])

*T1* before fentanyl injection (baseline), *T2* 2 min after fentanyl injection, *T3* 1 min after propofol or thiamylal injection, *T4* immediately before intubation, *T5* 2 min after intubation, *ETdes* end-tidal desflurane concentration, *BIS* bispectral index score, *HR* heart rate, *MAP* mean arterial pressure, *QTcD* heart rate-corrected QT interval dispersion

*p < 0.05 vs. T1 values or T4 values (in ETdes); #*p* < 0.05 vs. thiamylal group

**Fig. 1:**
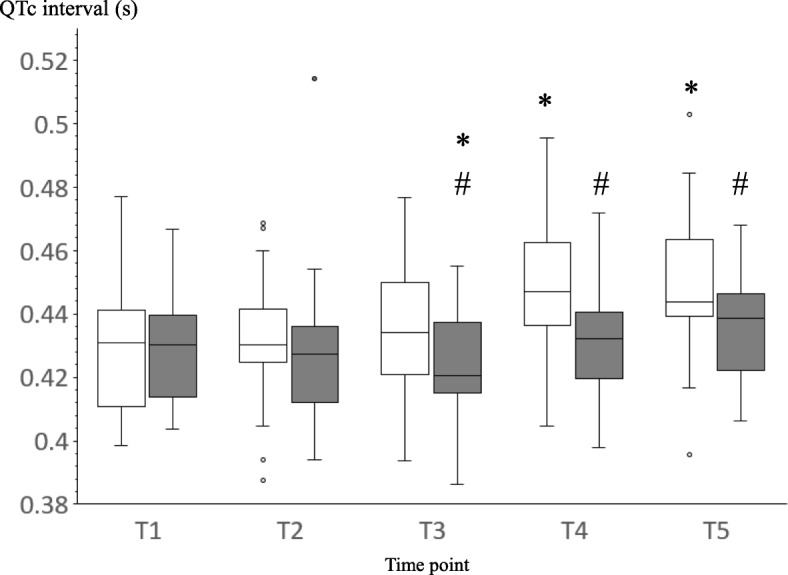
Heart rate corrected QT (QTc) interval in group thiamylal (white) and propofol (gray) at each time point. Values are expressed as median (line inside the boxes), IQR (boxes), and 10–90 percentiles (whiskers). T, group thiamylal; P, group propofol; T1, baseline; T2, after fentanyl injection; T3, 1 min after intravenous anesthetics injection; T4, 3 min after desflurane administration (immediately before intubation); T5, 2 min after intubation; *p < 0.05 vs. T1 values; #*p* < 0.05 vs. group thiamylal

Figure 1 shows the QTc interval values for both groups at each time point. The analysis of variance indicated significant effects of both intravenous agent and time. The interaction between the intravenous agent and the time point was also significant. These findings were confirmed by post hoc testing. In group P, the QTc interval value at T3 was significantly shorter than that at T1. In group T, the QTc interval values at T4 and T5 were significantly longer than that at T1. The QTc interval values at T3, T4, and T5 in group T were significantly longer than those in group P.

## Discussion

Propofol injection seemed to counteract the QTc interval prolongation associated with desflurane anesthesia induction, while neither intravenous anesthetic agent affected the QTcD.

Previous studies have shown that 6.0% desflurane significantly prolongs the QTc interval [1, 10]. It was more prolonged with 12% desflurane than 4% sevoflurane [5]. The differences in QT interval between two groups are small in our study. However, the duration of QTc prolongation at T4 and T5 compared to QTc values at T1 (baseline) were 20 (− 4, 44) and 20 (− 3, 43) ms in group T. The QTc interval exceeding 500 ms or prolongation of more than 20 ms from baseline increases the risk of TdP [11].

Furthermore, desflurane was associated with a high incidence of postoperative ventricular arrhythmias [11].

Although we found that propofol injection counteracted the QTc interval prolongation associated with desflurane anesthesia induction, our findings were inconsistent with those obtained by Kim et al. [12]. In their study, administration of desflurane at an inspiratory concentration of 6% after anesthetic induction with propofol 2 mg/kg prolonged the QTc interval [12]. These discrepancies might be explained by the following observations. First, our data on QT intervals were collected from averaged leads, whereas Kim et al. obtained their data from a single lead. Second, we evaluated the ETdes, whereas Kim et al. did not evaluate these values although they used the same desflurane concentration. We chose to use average values from the 12-lead ECG because of inter-lead variations in the QT interval [13]. Moreover, we previously found that propofol shortens the QTc interval [6, 14], and an injection of propofol counteracted the QTc interval prolongations associated with sevoflurane anesthesia induction [2] and antiemetic dose of droperidol [15].

Sympathetic stimulation is one of the factors interfering with the QTc interval. Direct laryngoscopy and intubation can cause this stimulation [16], and the effect of desflurane irritation on the respiratory tract mucosa has also been attributed to the release of catecholamines. The administration of desflurane at an inspiratory concentration of 1 MAC after anesthesia induction with propofol and 2 μg/kg fentanyl did not suppress tracheal intubation-induced QT prolongation [12]. However, in our study, premedication with 3 μg/kg fentanyl may have prevented the QTc interval prolongation to some extent by attenuation of the sympathetic stimulus during intubation. Chang et al. reported that pretreatment with 2 μg/kg fentanyl significantly diminished the QTc interval prolongation associated with laryngoscopy and tracheal intubation [17].

The changes in the QTc interval reflect the effects of the drugs and the depth of anesthesia on the ionic currents in cardiac myocytes. There are at least six distinct potassium currents in cardiac myocytes [18], with the main currents being the delayed rectifier (IK) and inward rectifier (IK1) currents. IK1 is the prime determinant of the resting conductance of cardiac myocytes, and IK is a key determinant of the action potential duration (APD). The IK current consists of a rapid component (IKr) and a slow component (IKs). Volatile anesthetics are known to cause IK current inhibition [4]. IK inhibition prolongs myocyte repolarization, thereby prolonging the QT interval. However, each volatile anesthetic affects the IKr and IKs channels differently, resulting in varying degrees of QT prolongation. Desflurane has been shown to inhibit IK currents, which is associated with significant lengthening of the APD, and the effects of desflurane primarily represented depression of IKs [19].

In contrast, it is important to note that the propofol concentrations required for IK suppression are higher than those currently used in clinical scenarios [20]. In addition, propofol predominantly suppresses L-type calcium currents (ICa) in a concentration-dependent manner and shortens the APD [21], and the QTc interval was shortened at a propofol concentration of 30 μM in rabbit myocytes [21]. The half maximal inhibitory concentration for the inhibitory effect of propofol on ICa was 9.8 μM [20]. The anesthetic effect of propofol is maintained in humans at blood concentrations ranging from 3.4 (19 μM) μg/mL to 4.5 (25 μM) μg/mL [22]. However, it is essential to consider its binding to blood proteins because this can reduce the free aqueous and effective concentration of propofol [23]. If protein binding is taken into account, the clinically relevant concentration of propofol is less than 2 μM [24]. However, a previous review article showed that propofol inhibits the ICa even at clinical concentrations (1 μM) [20]. Blockage of the ICa is known to attenuate the QT interval-prolonging effects of many drugs [25].

Previous studies have shown that QTcD is increased in patients with ischemic heart disease [26, 27] and that QTcD may be a predictor of dysrhythmic events in patients with congestive heart failure [28]. However, there have been conflicting reports on the QTcD-prolonging effects of desflurane [1, 5]. Our previous studies showed that propofol or thiamylal with sevoflurane does not affect QTD in similar study populations [2, 14]. Altogether, these studies suggest that co-existing cardiac disease could influence QTcD during desflurane anesthesia induction.

The present study had several limitations. Although manual measurement using a digitizer is the standard method to assess the QT interval, we used QT automatic analysis software. This software shows superior reproducibility and a few differences from manual measurements [29]. Although we did not prove that 5 mg/kg thiamylal was equipotent to 1.5 mg/kg propofol, our previous study showed that these doses of thiamylal and propofol decreased BIS to the same level during total intravenous anesthesia induction using them [2]. Although desflurane is known to prolong the QT interval significantly [11, 30], we never actually measured the QT interval during anesthesia induction in a desflurane-alone study population. However, desflurane is not generally administrated as the sole agent for anesthesia induction because of its potential to cause airway irritation. Furthermore, ETdes had not been maintained at a stable level. However, ETdes at T4 was measured during ventilation by face mask, on the other hand that at T5 was measured during ventilation by endotracheal tube.

## Conclusions

The present study showed that propofol, but not thiamylal, counteracted the QTc interval prolongation during anesthesia induction with desflurane. On the other hand, the use of propofol and thiamylal during anesthesia induction with desflurane did not affect the QTcD. Thus, we believe that propofol is more suitable than thiamylal for anesthesia induction with desflurane in patients with risk factors for ventricular dysrhythmia.

## Abbreviations

APD: Action potential duration
BIS: Bispectral index score
ECG: Electrocardiogram
ETdes: End-tidal desflurane concentration
HR: Heart rate
ICa: L-type calcium currents
IK: Delayed rectifier potassium current
IK1: Inward rectifier potassium current
IKr: Rapid component of IK
IKs: Slow component of IK
MAC: Minimum alveolar concentration
MAP: Mean arterial pressure
QTc interval: Heart rate-corrected QT interval
QTcD: QTc dispersion
QTD: QT dispersion
TdP: Torsade de pointes
